# A Case of Fistulous Opening of the Lung: Clinical Lectures in New York

**Published:** 1847-06

**Authors:** B. F. Wendel

**Affiliations:** Murfreesboro’, Tennessee; New York


**Art. II.**—*A Case of Fistulous Opening in the Lung: Clinical Lectures in New York.* By B. F. Wendel, M.D., of Murfreesboro', Tenn.

The following is a case of fistulous opening in the lung, the particulars of which were related to me by Dr. Sayre, of this city, under whose care the case now is:

A private, aged 38 years, while engaged, on the 9th of May last, in the action of Resaca de la Palma, received a bullet wound. The ball entered between the third and fourth ribs of the right side, an inch and a half or two inches to the right of the sternum, and, passing upwards and inwards, lodged under that bone near where it is joined by the third rib. Besides this wound, he received various injuries after falling. The ball was removed on the field, four hours after the injury, without much trouble; but little blood was lost. Erysipelatous inflammation soon set in, and this gave rise to a diffuse swelling, which was opened, and discharged about a quart of fetid pus; after which, during expiration, he discovered that air passed through the wound. His general health failed, hectic fever came on, and he remained in the hospital until the 1st of November, when the wound cicatrized, leaving a fistulous opening of the size of a goosequill, communicating with the lung, which still exists. Much of the lung is now impervious to air, gives no vesicular murmur, and is dull on percussion. There is now no discharge at the wound, but when Dr. Sayre's attention was first called to the case, a frothy mucus was emitted. The air passes through the opening with a sharp and loud noise, and with such force that it will extinguish a candle two feet distant. I saw this patient some two weeks since, and when my finger was placed over the opening, he spoke with comparative ease and force; removing the finger, his voice again became feeble. The matter is under consideration, whether the opening shall be closed.

The following is a case of *idiopathic peritonitis*, of which I cannot get the dates accurately, but report the most essential features as communicated to me by Dr. Sayre: A man of middle age, moderate circumstances, and temperate habits, by trade a ship-builder, was seized on Sunday, while in his ship-yard, with great pain in the abdomen, which he supposed to be colic. Sitting down on a log, he made firm pressure over the seat of the pain, and in a short time was relieved. The Friday following he had a slight chill while engaged in his occupation. The next Sunday he had a chill, and his wife called in medical aid; but he treated the physician rudely, and would not take the prescription, saying there was nothing the matter with him. Being at a dinner party the next Thursday, his wife observed that he did not eat as much as usual, that his countenance was pale, and that he frequently made pressure over his abdomen. Starting home, he was seized with a severe chill, and told his wife that he would leave her and run home; on getting there, a physician was sent for. The physician found him shaking with a severe chill, with pallor of the countenance, a frequent, small, weak pulse, and some tenderness over the abdomen. He was cupped and blistered over the tender part, but it was observed that his pulse became frequent and feeble in proportion to the quantity of blood taken. A dose of calomel and Dover's powder was ordered to be taken at bed-time, to be followed by oil in the morning. The physician not supposing the case to be serious, did but little more. The following Tuesday the patient died.

*Post mortem appearances.*—The abdominal cavity seemed to be a mass of lymph. The intestines were bound together by continuous bands of recently effused lymph, and the peritoneum lining the abdominal wall was literally covered with the same. All the other organs were healthy.

*Professor Parker's Clinic*, Monday, April 26th.—Case I.—The first case presented was one of common varus, occurring in a healthy child, about three months old. I mention this to bring up another malformation in the same child: It had no penis, externally, though the rudiments of the organ could be felt under the skin and pushed out to some extent. The testes were perfect and well developed. Prof. P. remarked, that he had never before seen such a case. The mother attributed the malformation to a fright from a bull during the third month of gestation.

Case II.—*Enlargement of the cervical lymphatic ganglia.*—Suppuration had taken place, giving rise to a swelling in the cervical region. Has pain in the head and knees, and his urine deposits a copious sediment. The swelling was laid freely open and ordered to be dressed with lint and cataplasm.

Case III.—A man aged 30 years, of intemperate habits and strumous diathesis, next came up, complaining of two sores on his sternum. On examination, it was found that, in addition to the sores, there was necrosis of the upper end of the sternum, and perhaps also of the clavicle. He had labored under the venereal disease several times, and this may have been the exciting cause of the disease of the bones. In the right lung tubercles had been deposited, producing feebleness of respiration on that side, and in the left lung puerile respiration and bronchophony were heard. In addition to all this, there was curvature of the spine to the right and left sides.

*Prognosis.*—Of course this is unfavorable, for the patient is scrofulous, intemperate, has had venereal disease several times, and, most probably, has taken mercury.

*Treatment.*—But little can be done. Good diet, warm bath and friction.

Case IV.—*Periostosis, or chronic inflammation of the lower end of the tibia and fibula, not invading the joint.*—The subject of this case is of a scrofulous diathesis. The joint admits of but little motion, but gives no roughness when moved. Apprehensive that the joint will become involved and amputation have to be resorted to.

*Treatment.*—Good diet and good air. Locally, wash and dress with salt water poultice.

CASE V.—*Burrowing ulcer, of four years' standing, under the fascia of the forearm.*—Has been treated various ways.

*Treatment.*—Lay freely open, stuff with lint, and when inflammation occurs, apply a poultice.

CASE VI.—A young lady presented herself with inability to flex and extend the forearm, save to a limited extent. She stated that, two or three years since, her hand was squeezed by a young man, and that the pressure at the time gave her some uneasiness; that ten months ago she was bled, and that in two or three days after pretty severe inflammation occurred in the arm. Prof. P. remarked, that the squeeze could not be referred to as the cause of this adhesion between the muscles; that the bleeding did not cause it, for the inflammatory symptoms did not occur for three days after the operation, whereas if a nerve had been pricked, the symptoms would have followed immediately. The girl stated that she had gradually been regaining the use of her arm; and the professor remarked that the *tinctura temporis* was the remedy in such cases.

*Nevis materni, or erectile tumor.*—The tumor was in a child three months of age, and was situated on the forehead. The mother said she did not observe it until some time after its birth, but its nidus must have been there. The tumor varies in color and temperature as it happens to be composed in chief part of arteries or veins.

The true mode is to take such tumors in time. The methods of treatment are, the hot needles, the thread, ligation, etc. The hot needles or the actual cautery are the best. In this case the hot needles were used.

*Varicose vein of the left lower extremity.*—Treatment is either palliative or radical. As the patient will not now submit to an operation, the shower bath and roller were ordered. Prof. P.'s radical treatment is to pass a needle under the vein, cut down to it, and pass the twisted suture over it.

A tailor, 26 years old, of regular habits, presented himself, complaining of a cough under which he had labored for a long time; but he said it had not annoyed him until six weeks since; says he raises much when he coughs; is nervous, and has palpitations, night sweats, and is losing flesh daily. Most of his relations have died of disease of the lungs. The left side of the thorax is more depressed than the right, and, during inspiration, the right lung is observed to fill with air more completely than the left. These facts lead us to suspect the left lung. Percussion reveals less resonance in the left than in the right, though the right is somewhat dull. Auscultation gives us prolonged and bronchial respiration, as is the case when tubercles are not far advanced. Tubercles in both lungs plainly, but especially in the left. But little can be done in such a case.

*Tertiary syphilis.*—A young woman came up, complaining of pain in the limbs, commencing about sunset. Prof. Parker observed, that a great diagnostic mark between true and syphilitic rheumatism was found in the time when the pains occurred. In true rheumatism they begin in the evening, or after the patient has got warm in bed; while those of syphilitic rheumatism commence about four or five o'clock in the evening.

Dr. Fell, of this city, reports a number of cases of tetanus occurring in his practice, cured by strychnia. His plan is to commence with from one-twelfth to one-eighth of a grain of strychnia, and gradually increase it until slight twitchings of the muscles are produced.

A new plan of treating syphilis, as practised by Dr. Anderson, of Staten Island hospital, is under trial at Bellevue. It is this: Large doses of some of the preparations of mercury twice a day, worked off in the morning; say a scruple to half a drachm of calomel, night and morning, and worked off by some laxative. This is said to be the quickest mode of cure, (curing some in a week,) and as effectual as any other.

I saw, two weeks ago, a case of amputation of the arm at the shoulder, in which the "letheon" was administered. The ether rendered the patient stupid, but he grunted and appeared to suffer much pain. Indeed, the ether has been frequently administered at this institution, and thus far with but little success.

A day or two since I heard a medical student extolling a quack in this city, who has gained a great reputation for the cure of *prolapsus uteri*. The student declared that he had seen cases cured by his method. And what, would you guess, is his potent remedy? No other than *a quart of hazlenuts* finely powdered, shells and all, boiled in a quart of milk down to a pint, of which the patient is to take a teaspoonful three times a day!

New York, May 1, 1847.

